# Snakebites in Rio Branco and surrounding region, Acre, Western Brazilian Amazon

**DOI:** 10.1590/0037-8682-0214-2020

**Published:** 2020-09-25

**Authors:** Laiane Parente de Oliveira, José Genivaldo do Vale Moreira, Jacqueline de Almeida Gonçalves Sachett, Wuelton Marcelo Monteiro, Dionatas Ulises de Oliveira Meneguetti, Paulo Sérgio Bernarde

**Affiliations:** 1 Universidade Federal do Acre, Programa de Pós-Graduação Stricto Sensu em Ciências da Saúde na Amazônia Ocidental, Rio Branco, AC, Brasil.; 2 Universidade Federal do Acre, Centro Multidisciplinar, Cruzeiro do Sul, AC, Brasil.; 3 Universidade do Estado do Amazonas, Manaus, AM, Brasil.; 4 Fundação Alfredo da Matta, Diretoria de Ensino e Pesquisa, Manaus, AM, Brasil.; 5 Fundação de Medicina Tropical Dr. Heitor Vieira Dourado, Manaus, AM, Brasil.; 6 Universidade Federal do Acre, Colégio de Aplicação, Rio Branco, AC, Brasil.; 7 Universidade Federal do Acre, Campus Floresta, Centro Multidisciplinar, Laboratório de Herpetologia, Cruzeiro do Sul, AC, Brasil.

**Keywords:** Ophidism, Snake bite, Epidemiology, Amazon

## Abstract

**INTRODUCTION:**

Snakebites are considered a neglected tropical disease in many countries in Latin America, including Brazil. As few studies have assessed snakebites in the Amazon region and especially in the state of Acre, epidemiological studies are of great importance. The present study aimed to describe the epidemiological characteristics of snakebites in the Rio Branco region, observing their characteristics in rural and urban areas and their correlation with rainfall and river outflow.

**METHODS:**

This retrospective, descriptive study analyzed epidemiological information obtained from snakebite notifications registered on the Information System for Notifiable Diseases that occurred from March, 2018 to February, 2019. The cases of snakebite were correlated with rainfall and flow.

**RESULTS:**

A total of 165 cases of snakebite were registered in the period. Most cases were caused by *Bothrops* and affected mainly individuals of the male sex who were between 21 and 30 years old. Most of the snakebites occurred in Rio Branco (71.52%; 29 cases per 100,000 inhabitants). Of these, 60.2% occurred in the urban area and 39.8% in the rural area and the majority occurred during the rainy season.

**CONCLUSIONS:**

Although studies have shown that a majority of cases occur in rural areas, in this study, urbanization of snakebites was observed. The *Bothrops* genus was responsible for the highest number of snakebites and, during the rainy season, bites occurred more frequently. Educational prevention campaigns, population advice, and first aid in case of snakebites for the population are thus suggested.

## INTRODUCTION

Snakebites are considered a neglected tropical disease in many countries in Africa, Asia, and Latin America^1^. Estimates show that more than 5 million snakebites occur every year in the world, with approximately 2 million envenomations which result in 94,000 deaths^2^. This information on the estimation of cases per snakebite may be underestimated when the existence of non-notified cases is considered, and thus may represent data exceeding those mentioned above^3^.

In Brazil, there are 405 known species of snakes^4^. Of these, sixty-six venomous snakes belong to the families Elapidae (genera: *Micrurus*, and *Leptomicrurus*), and Viperidae (genera: *Bothrocophias*, *Bothrops*, *Crotalus*, and *Lachesis*)^4^. Most of the snakebites recorded in the Amazon are caused by *Bothrops* (88.7%), with *B. atrox* being responsible for most cases of envenomation^3^ ^,^ ^5^ ^,^ ^6^.

In the state of Acre, the region known as Alto Juruá is characterized by a high incidence of snakebites^6^ ^-^ ^8^. Pierini et al.^7^ found a prevalence of 13% for cases of snakebites in traditional populations (extractivists, riverines, and indigenous). In this region, the prevalence of snakebites is associated mainly with the activities of agriculture and extractivism, which are practiced by people living in rural areas and forests and who are thus more vulnerable^6^ ^-^ ^10^.

In a retrospective study conducted at the Regional Hospital of Juruá in Cruzeiro do Sul in the state of Acre, Bernarde and Gomes^8^ recorded the occurrence of 195 cases of snakebites attended to at the hospital in the two-year period (August, 2007 to July, 2009). In the municipality of Tarauacá, also in the state of Acre, a retrospective study^12^ showed a high coefficient of morbidity due to snakebites in 2016 (72.5 cases per 100,000 inhabitants), which is higher than the coefficients recorded in municipalities such as Cruzeiro do Sul and Rio Branco. Although it is considered a health problem, there is still a knowledge gap regarding snakebites and their relationship with environmental conditions in the region of Rio Branco. The last study carried out in the region was conducted in 2002 and only contemplated epidemiological and clinical characteristics^3^. In this respect, the present study aimed to describe not only the epidemiological and clinical characteristics of snakebites in the region of Rio Branco, Acre, Western Amazonia, but also the characteristics of the accidents that occurred in rural and urban areas and their correlation with rainfall and low seasonal river levels.

## METHODS

The study was carried out using a retrospective approach and focused on the epidemiology of snakebites which occurred in the period from March, 2018 to February, 2019 and on the correlation of these cases with the respective rainfall and low seasonal river levels, the period from 2009 to 2018 was used.

**FIGURE 1:**
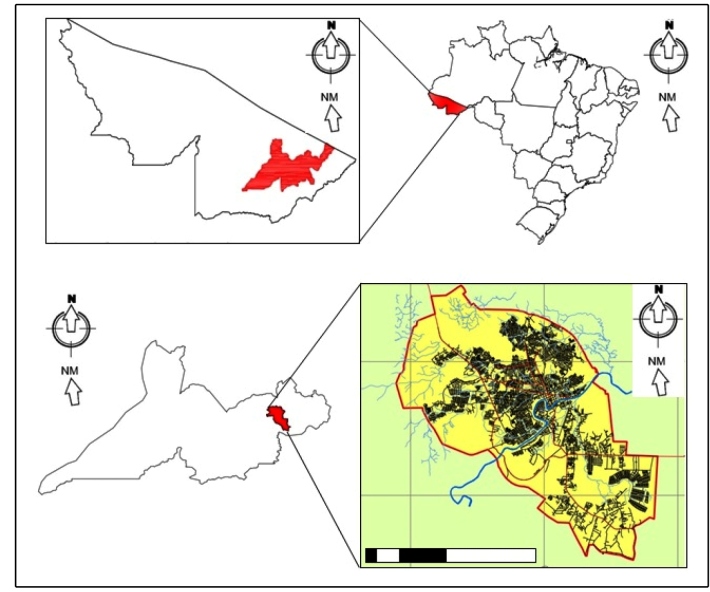
Map of Brazil and State of Acre and location of the municipality of Rio Branco (above) and the delimitation of its urban area (below).

The epidemiological data obtained were collected from the SINAN (Health Information Notification System) registry in the epidemiological surveillance sector for the municipality of Rio Branco, Acre, and included the cases dealt with in the accident and Emergency Hospital of Rio Branco. Accident and Emergency Hospital Rio Branco, which is the reference Center for the treatment of cases of snakebites occurring in the region of Acre Vale. This hospital treats victims of snakebites mainly from Rio Branco (Figure 1), but may also receive patients from other nearby municipalities (Assis Brasil, Bujari, Capixaba, Plácido De Castro, Porto Acre, Senador Guiomard, Sena Madureira and Xapuri) or further away (e.g., Boca do Acre, Jordão, Tarauacá and even from Porto Velho, state of Rondônia). The municipality of Rio Branco, Acre, has an estimated population of 407,319^12^, its territory is 8,834,942 km^2^ and it is located in northwestern Brazil. According to the city plan, it can be observed that over the years, there has been an urban growth which has invaded the surrounding rural areas^13^. However, despite this urbanization, some road systems located inside the urban area of Rio Branco still have characteristics of the old rural area (Figure 1).

From the Health Information Registry, the following variables were obtained: year and month of occurrence, the identification of the snake type (type of snakebite), location of accident (urban or rural) in the municipality of occurrence, and data on socio-demographics such as age and gender, in addition to clinical data such as the anatomical region affected, the time that had elapsed between the accident and care being administered, the number of vials used, local and systemic manifestations, the type of serum used in the treatment of victims and the severity of the envenomation. The region’s rainfall data and seasonal river levels were obtained from the sites of the National Water Agency - ANA^14^ and the National Institute of Meteorology - INMET^15^. 

The ratio of morbidity rates (per 100,000 inhabitants) was calculated by dividing the number of people who had suffered snakebites by the number of people living in the region during the period, multiplied by 100,000. Only the cases that occurred in the municipality of Rio Branco were considered, since some of the cases may have been treated in the municipalities of origin and not in the Accident and Emergency Hospital in Rio Branco. With respect to the correlation of rainfall, seasonal river levels, and snakebites, during the period from 2009 to 2018, the null hypothesis (H_0_), that there is no statistically significant correlation (p < 0.05) between the variables in focus, was tested using the Spearman non-parametric test, since a previous check linked the acceptance of the assumptions with the analysis of the bias of the parametric statistics^16^ ^,^ ^17^.

 This research is part of the project named "Snakebites occurring in Rio Branco and region (Acre)" approved by the Committee on Ethics and Research with Human Subjects at Fundação de Medicina Tropical Dr. Heitor Vieira Dourado (approval no. 3.223.051).

## RESULTS

In the period from March, 2018 to February, 2019, 165 cases of snakebites were recorded, the majority (76.36%) were classified as being caused by *Bothrops*, followed by non-venomous snakes (9.70%), *Micrurus* (1.82%), and *Lachesis* (1.21%). Many of the snakebites occurred in the municipality of Rio Branco (71.52%; 29 cases per 100,000 inhabitants). Snakebites were almost as common in the urban area (46.06%) as the rural area (53.94%) and a greater proportion occurred during the rainy season (64.24%) (Table 1).

**TABLE 1:** Clinical and epidemiological characteristics of the cases of snakebites occurring in Rio Branco and the surrounding region (Acre - Brazil) during the period from March, 2018 to February, 2019.

Characteristics (n = 165)	n	%
Snake genus		
*Bothrops*	126	76.36%
*Lachesis*	2	1.21%
*Micrurus*	3	1.82%
Non-venomous / dry bite	16	9.70%
Ignored	18	10.91%
Season		
Rainy (November to April)	106	64.24%
Dry (May to October)	59	35.76%
Sex		
Male	107	64.85%
Female	58	35.15%
Municipality of occurrence		
Rio Branco	118	71.52%
Bujari	10	21.28%
Porto Acre	7	14.89%
Senador Guiomard	5	10.64%
Sena Madureira	5	10.64%
Capixaba	4	8.51%
Assis Brasil	2	4.26%
Jordão	2	4.26%
Tarauacá	2	4.26%
Xapuri	1	2,13%
Plácido de Castro	1	2.13%
Boca do Acre	4	8.51%
Porto Velho	4	8.51%
Area of occurrence		
Rural	89	53.94%
Urban	76	46.06%
Age group, years		
0 to 10	25	15.15%
11 to 20	33	20%
21 to 30	37	22.42%
31 to 40	28	16.97%
41 to 50	21	12.73%
51 to 60	11	6.67%
> 60	10	6.06%
Occupation		
Farmer	35	21.21%
Student	53	32.12%
Housewife	25	15.15%
Other	52	31.52%
Time to hospital care		
0 to 1 hour	37	22.42%
1 to 3 hours	48	29.09%
3 to 6 hours	26	15.76%
6 to 12 hours	5	3.03%
12 to 24 hours	12	7.27%
> 24 hours	30	18.18%
Ignored	7	4.24%
Anatomical region of the bite		
Foot	91	55.15%
Leg	46	27.88%
Hand	14	8.48%
Arm	5	3.03%
Head	4	2.42%
Thorax	1	0.61%
Ignored	4	2.42%
Classification of envenomation		
Light	120	72.73%
Moderate	36	21.82%
Severe	9	5.45%
Local manifestations and complications		100%
Pain	152	92.12%
Edema	120	72.73%
Bruising	3	1.82%
Cellulitis	1	0.61%
Necrosis	1	0.61%
Systemic manifestations and complications		
Change in clotting time (performed clotting exams, n = 38)	9	23.68%
Headache	5	3.03%
Bleeding	4	2.42%
Nausea	2	1.21%
Paresthesia	2	1.21%
Odynophagia	1	0.61%
Angioedema	1	0.61%
Hemorrhagic cerebrovascular stroke	1	0.61%
Dyspnea	1	0.61%
Eyelid ptosis	1	0.61%
Decreased level of consciousness	1	0.61%
Somnolence	1	0.61%
Dizziness	1	0.61%
Blurred vision	1	0.61%

Regarding the distribution of accidents, it was observed that the cases of snakebites occurred more often among males (64.85%) aged 21 to 30 years (22.42%). The main occupations of the victims were students (32.12% of the cases) and farmers (21.21%). 

The largest proportion of the victims (29.09%) was treated in the hospital within the first three hours after the accident. However, 18.18% received late care, and were treated after 24 hours and most of the cases were classified as light (77.73%).

The lower limbs, feet (55.15%) and legs (27.88%) were the most affected. Local manifestations and complications consisted mainly of pain (92.12%) and edema (72.73%). Other manifestations also occurred, such as headache (3.03%), paresthesia (1.21%) and nausea (1.21%). Systemic manifestations and complications, such as changes in blood clotting time (23.68%), bleeding time (2.42%), dyspnoea (0.61%), palpebral ptosis (0.61%), haemorrhagic cerebrovascular stroke (0.61%) and decreased level of consciousness (0.61%) were also observed. During the study period no deaths were recorded.

Compared to the region as a whole, 71.52% of the cases of snakebites occurred in the municipality of Rio Branco. Of these cases, 60.17% occurred in the urban area and 39.83% in the rural area (Table 2). Snakebites caused by *Bothrops* were frequent both in the urban area (74.65%) and in the rural area (78.72%); there was no record of the occurrence of a snakebite caused by *Lachesis* in the urban area; and in the rural area there were no recorded bites caused by *Micrurus*.

**TABLE 2:** Comparison of clinical and epidemiological manifestations of snakebites occurring in urban and rural areas in the municipality of Rio Branco (Acre, Brazil) during the period from March, 2018 to February, 2019.

Characteristics (n = 118)	Urban	Rural
	71 (60.17%)	47 (39.83%)
Snake genus		
Bothrops	53 (74.65%)	37 (78.72%)
Lachesis	0	2 (4.26%)
Micrurus	1 (1.41%)	0
Non-venomous / dry bite	8 (11.27%)	4 (8.51%)
Ignored	9 (12.68%)	4 (8.51%)
Season		
Rainy (November to April)	45 (63.38%)	36 (76.6%)
Dry (May to October)	26 (36.62%)	11 (23.4%)
Sex		
Male	41 (57.75%)	33 (70.2%)
Female	30 (42.25%)	14 (29.8%)
Age group, years		
0 to 10	17 (23.94%)	4 (8.5%)
11 to 20	13 (18.31%)	15 (31.91%)
21 to 30	10 (14.08%)	9 (19.15%)
31 to 40	14 (19.72%)	8 (17.02%0
41 to 50	10 (14.08%)	6 (12.77%)
51 to 60	5 (7.04%)	1 (2.13%%)
> 60	2 (2.82%)	4 (8.51%)
Occupation		
Farmer	2 (2.82%)	14 (29.79%)
Student	27 (38.03%)	18 (38.30%)
Housewife		
Other	42 (59.15%)	15 (31.91%)
Time to hospital care		
0 to 1 hour	24 (33.80%)	12 (25.53%)
1 to 3 hours	23 (32.39%)	12 (25.53%)
3 to 6 hours	9 (12.68%)	6 (12.77%)
6 to 12 hours	2 (2.82%)	3 (6.38%)
12 to 24 hours	7 (9.86%)	1 (2.13%)
> 24 hours	4 (5.63%)	9 (19.15%)
Ignored	2 (2.82%)	4 (8.51%)
Anatomical region of the bite		
Foot	41 (57.75%)	25 (53.19%)
Leg	22 (30.1%)	13 (27.66%)
Hand	3 (4.23%)	5 (10.64%)
Arm	2 (2.82%)	1 (2.13%)
Head	0	1 (2.13%)
Thorax	1 (1.41%)	0
Ignored	2 (2.82%)	2 (4.26%)
Classification of envenomation		
Light	63 (88.73%)	33 (70.21%)
Moderate	7 (9.86%)	12 (25.53%)
Severe	1 (1.41%)	2 (4.26%)
Local manifestations and complications		
Pain	65 (91.54%)	43 (91.49%)
Edema	49 (69.01%)	34 (72.34%)
Bruising	0	1 (2.13%)
Necrosis	1 (1.41%)	0
Compartment syndrome	0	0
Manifestations and systemic complications		
Headache	1 (1.41%)	1 (2.13%)
Paresthesia	2 (2.82%)	0
Angioedema	1 (1.41%)	0
Nausea	1 (1.41%)	0
Dizziness	0	1 (2.13%)
Change in clotting time	2 (9.09%)	2 (22.22%)
Bleeding	0	1 (2.13%)

In relation to the historical series of snakebites (2009 to 2018), it has been found that the highest number of snakebites tends to concentrate in the months when the highest average precipitation and lowest river level occur. In the Spearman’s correlation analysis, there was a positive correlation between the variable snakebites with seasonal river level (*r* = 0.61, n = 120, p < 0.01) and with precipitation (*r* = 0.30, n = 120, p < 0.01). There was also a positive correlation between seasonal river level and precipitation (*r* = 0.49, n = 120, p < 0.01).

Snakebites occurred mainly in males, and a higher proportion of cases in females occurred in the urban area (42.5%) compared to rural areas (29.8%) (Pearson’s chi-squared = 5.8182, p = 0.0159). The 0-10 year age group (23.94%) in the urban area was the most affected; and in the rural area the 11-20 year age group was the most prevalent age group (31.91%). In the urban area, the majority of victims of snakebites had “other” listed as their occupation (59.15%), while in rural areas most of the victims were students (38.30%) and farmers (29.79%) (Table 2). 

In the urban area, the largest proportion of the victims (66.19%) were treated in the hospital in the period up to 3 hours after the accident; however, in the rural area, the proportion of care given in the same period was 51.06%. The highest percentage of mild cases was registered in the urban area (88.73%) and moderate and severe in rural areas (25.53% and 4.26%, respectively) (Pearson chi-squared = 11.375, p = 0.0034).

## DISCUSSION

During a period of 12 months, 165 cases were recorded in the Rio Branco region, more than that reported for the municipality of Tarauacá (29 cases) by Saboia and Bernarde^11^ and for the Cruzeiro do Sul region (133 cases) by Mota-da-Silva et al.^6^. However, a lower morbidity ratio was observed in this study for the city of Rio Branco (29 cases/100,000 inhabitants) when compared to Tarauacá (72.5 cases/100,000 inhabitants) and Cruzeiro do Sul (76.2/100,000 inhabitants). This is probably due to a larger population who live in rural areas and forests and agricultural and extractive activities in these last two regions^6^ ^,^ ^11^, in relation to the capital. In relation to the previous study conducted in Rio Branco by Moreno et al.^3^, which recorded 89 cases, a reduction in the number of cases per inhabitants was observed, from 35.1/100,000 inhabitants, to 29/100,000 inhabitants. This reduction may be associated with the urbanization process of accidents, which are now more frequent in this area.

Most of the registered accidents were attributed to *Bothrops* (76.36%), which corroborates information in the literature which states that this type of envenomation is the most frequent in the Amazon^6^ ^,^ ^18^ ^,^ ^19^. As expected, envenomation by *Micrurus* and *Lachesis* were uncommon (less than 2% each), and corroborates findings by other authors for the same region^5^ ^,^ ^6^ ^,^ ^19^ ^,^ ^20^. There was no record of an envenomation involving *Crotalus* in this study. This was expected, since the rattlesnake (*Crotalus durissus*), is not reported in Acre^8^, since it inhabits areas of cerrado, a type of vegetation that is absent in the state of Acre^21^.

The snakebites analyzed in the present study had a greater occurrence during the rainy season (64,24%) and the historical series (2009-2018) was statistically associated with the low seasonal river levels and rainfall, thus confirming the observations of other studies^3^ ^,^ ^6^ ^,^ ^8^ ^,^ ^11^ ^,^ ^22^ ^-^ ^24^. This predominance of snakebites in the wettest months is probably related to the increase in snake activity in this period, due to the greater availability of prey and reproductive activities^25^ ^,^ ^26^. In addition, a greater chance of encounters between snakes and humans can be due to certain agricultural and extractive activities developed in this period, and the fact that flooding causes these animals to search for dry land areas^3^ ^,^ ^6^ ^,^ ^27^. 

The fact that the higher frequency of victims were male and bitten on the lower limbs corresponded to the epidemiological profile observed for the Brazilian Amazon^5^ ^,^ ^6^ ^,^ ^8^ ^,^ ^19^, as well as for the other regions of Brazil^28^.

In regard to the locations of the accidents, it is noted that, in general, the rural areas showed slightly more than half of the cases (53.94%), and when analyzing only the municipality of Rio Branco, there was a greater predominance in the urban area (60.17%), contrary to what was observed in other studies, in which there is a greater predominance of snakebites in rural areas (more than 85% of the cases)^5^ ^,^ ^6^ ^,^ ^11^ ^,^ ^19^. This urbanization of snakebites was also observed for Belém do Pará in the eastern Amazon^29^, where 66% of cases were recorded in the urban area. This situation may have occurred due to the demographic and spatial growth of the city of Rio Branco, the decentralization of the urban area^30^, disordered expansion, and exodus from rural to urban areas^31^, where more than 90% of the population is concentrated^12^. There is also the existence of families living in high risk areas on the banks of the Acre River and associated streams, which are vulnerable to flooding. There is also occupation in the form of invasions of permanent preservation areas close to Environmental Protection Areas^31^ ^-^ ^33^, which may also contribute to these accidents. Another factor that can contribute to snakebites in urban areas are the green areas (forest fragments) and floodplains present in cities, which favor the occurrence and encounter of some species of snakes^29^ ^,^ ^34^. The city of Rio Branco has some patches of forest and rivers^35^ ^,^ ^36^, which may explain some of the snakebites in the urban area.

Analyzing the time elapsed from the moment of the snakebite to the time of hospital care, most of the victims received care in the first six hours (67.27%), which indicates care occurring earlier when compared to the previous study by Moreno et al.^3^. Their study indicates that only 58.3% of the cases were treated in this period. This decrease in the “time to treatment” was also described in a study carried out by Mota-da-Silva et al.^6^ in the Alto Juruá region (60% treated within six hours), who attributed the cause to the increase in the number of ambulances and improvements in roads and telephone services as possible factors for this improvement in time.

A marked and expected difference was the greater speed of care of cases occurring in the urban area, which were closer to the hospital units than those in the rural area. In the urban área, 78.87% of the cases received care in the first six hours and in the rural area 63.83%. For the 24-hour period after the accident, the proportion was lower in the urban area (5.63%) and higher in the rural area (19.15%). This demonstrates that in the Amazon, some cases of snakebites occur in locations so far from urban centers, that they result in delays in hospital care or even lack of access to antivenom^7^ ^,^ ^27^. These are often due to displacement difficulties and, as such, may contribute to the severity of cases^9^ ^,^ ^37^. In the urban area, there was a predominance of moderate and severe cases in the rural area, which is probably associated with greater speed of care.

Regarding the severity of snakebites, the frequency of mild (72.73%), moderate (21.82%) and severe (5.45%) corroborates with the study by Mota-da-Silva et al.^6^ conducted in the Alto Juruá region (50%, 36.5%, and 13.5%, respectively) which shows that the highest occurrence of accidents was mild, followed by moderate and severe. However, these findings differ from what was observed in the previous study conducted in Rio Branco in 2002, in which moderate cases (48.6%) accounted for the majority of accidents, followed by mild (31.2%) and severe (20.2%) cases^3^. This may be due to an improvement in the structure of the health care network in the state of Acre with serum availability and training of professionals for more appropriate clinical management of snakebites. 

The most frequent symptoms presented in this study were pain (92.12%) and edema (72.73%), which are the most commonly observed in cases of *Bothrops* envenomations^3^ ^,^ ^6^ ^,^ ^11^ ^,^ ^38^. Other symptoms and even complications observed less frequently in this study, such as cellulitis, ecchymosis, hemorrahage and necrosis, are also observed in *Bothrops* envenomations^6^ ^,^ ^11^ ^,^ ^19^ ^,^ ^38^. The blood clotting time observed (23.68%) was lower than that recorded in the previous study by Moreno et al.^3^ (43.1%) for Rio Branco and Tarauacá (55.5%)^11^ and the Alto Juruá region (82.5%)^6^. This low frequency may have been due to the low sample number of tests performed, only 38, compared to that of the other three studies (90 to 129 tests performed)^3^ ^,^ ^6^ ^,^ ^11^.

Some differences were observed between the snakebites that occurred in the urban and rural areas of Rio Branco. Among them, the two laquetic accidents which occurred in the rural area and though none occurred in the urban area, which was to be expected, since the snake *Lachesis muta* is a species associated with the dense forests^39^. More cases attributed to non-venomous snakes or “dry bites” were present in the urban area, where it is more common to record accidents with species that are not of medical interest^34^. A higher proportion of cases among females occurred in the urban area compared to rural areas, which may be related to the fact that a large portion of the accidents in cities occur in households^34^ and in the rural area which is a more occupational setting, involving mostly farming activities and the extraction process which is carried out mainly by males^9^ ^,^ ^10^ ^,^ ^27^ ^,^ ^28^. This same hypothesis could explain the higher proportion of children (under 10 years old) bitten in the urban area (23.94%) than in the rural area (8.5%) in the present study.

Snakebites mainly affect adult males and tend to occur in the lower limbs. Although several studies have shown that most cases occur in rural areas, this study observed an urbanization of ophidism in the city of Rio Branco. The genus *Bothrops* was responsible for the highest number of snakebites and during the rainy season the accidents occur with a higher frequency, presenting a positive correlation with these events in the studied region. The performance of retrospective studies is important, among other reasons, because this information, when properly collected and recorded in the notification forms, becomes a valuable source of epidemiological data, and provides greater reliability and the possibility of better understanding about a given health problem. Educational snakebites prevention campaigns, population advice and first aid in case of this type of accidents for the populations of rural and urban areas are thus suggested.
